# Distinct clinicopathological features in metanephric adenoma harboring BRAF mutation

**DOI:** 10.18632/oncotarget.11117

**Published:** 2016-08-08

**Authors:** Anna Caliò, John N. Eble, Ondrej Hes, Guido Martignoni, Saul E. Harari, Sean R. Williamson, Matteo Brunelli, Adeboye O. Osunkoya, Lisha Wang, Eva Comperat, Antonio Lopez-Beltran, Mingsheng Wang, Shaobo Zhang, Kendra L. Curless, Kristin M. Post, Hsim-Yee Chang, Claudio Luchini, Lee Ann Baldrige, Gregory T. MacLennan, Rodolfo Montironi, David J. Grignon, Liang Cheng

**Affiliations:** ^1^ Department of Pathology and Laboratory Medicine, Indiana University School of Medicine, Indianapolis, Indiana, USA; ^2^ Department of Pathology, University of Verona, Verona, Italy; ^3^ Department of Pathology, Charles University Hospital Plzeň, Pilsen, Czech Republic; ^4^ Department of Pathology, Pederzoli Hospital, Peschiera, Italy; ^5^ Department of Pathology and Laboratory Medicine, Henry Ford Health System, Detroit, Michigan, USA; ^6^ Department of Pathology, Emory University School of Medicine, Atlanta, Georgia, USA; ^7^ Michigan Center for Translational Pathology, University of Michigan, Ann Arbor, Michigan, USA; ^8^ Department of Pathology, Groupe Hospitalier Pitié-Salpêtrière, Paris, France; ^9^ Unit of Anatomical Pathology, Department of Surgery, Faculty of Medicine, Cordoba, Spain and Champalimaud Clinical Center, Lisbon, Portugal; ^10^ Departments of Pathology and Laboratory Medicine, Case Western Reserve University, Cleveland, Ohio, USA; ^11^ Department of Pathological Anatomy and Histopathology, School of Medicine, Polytechnic University of The Marche Region (Ancona), Ancona, Italy

**Keywords:** kidney, metanephric adenoma, BRAF, nephroblastoma/Wilms tumor, immunohistochemistry

## Abstract

*BRAF* mutation recently has been reported in metanephric adenoma. We sought to determine the clinical and morphologic features of *BRAF*-mutated metanephric adenoma and to correlate *BRAF* mutation with BRAF V600E immunohistochemical staining results. A series of 48 metanephric adenomas and 15 epithelial-predominant nephroblastomas were analyzed for the occurrence of *BRAF* mutation (*BRAF* V600E/V600E complex, *BRAF* V600D, *BRAF* V600K and *BRAF* V600R) using the BRAF RGQ PCR kit (Qiagen). Immunohistochemistry was performed using monoclonal mouse antibodies against p16^INK4^ and VE1 (Spring Bioscience), recognizing the BRAF V600E mutant protein. Forty-one of 48 cases (85%) showed *BRAF* V600E mutation; none of the other *BRAF* variants was detected. Of 41 *BRAF*-mutated metanephric adenomas, 33 showed positive VE1 immunostaining (sensitivity 80%, specificity 100%); in all cases we detected p16^INK4^ expression regardless of *BRAF* mutation status. All epithelial-predominant nephroblastomas were *BRAF*-wild-type and none expressed VE1. The following features were associated with *BRAF* V600E mutation: older patients (p=0.01), female predominance (p=0.005) and the presence of a predominantly acinar architecture (p=0.003). In summary, *BRAF*-mutated metanephric adenomas were associated with older age, female predominance, and the presence of a predominant acinar component. A subset (20%) of *BRAF*-mutated metanephric adenomas was not detected by VE1 immunostaining.

## INTRODUCTION

Metanephric adenoma of the kidney is an uncommon benign neoplasm which is usually asymptomatic and discovered incidentally. These tumors mostly occur in middle-aged individuals, with a female predominance (2:1), although the age distribution is broad, ranging from children to the elderly [1–4]. On gross examination, metanephric adenomas are typically circumscribed, not-encapsulated, solid masses. Histologically, these neoplasms are typically composed of small epithelial cells arranged as tightly packed small acini. A hyalinized or edematous stroma is present occasionally. Psammoma bodies are common. The cells have scant cytoplasm, round nuclei, and variably-present nuclear grooves. However, metanephric adenomas may assume a variety of architectures and may thus present a diagnostic challenge to the pathologist. The main differential diagnostic considerations for metanephric adenoma are epithelial-predominant nephroblastoma in children and the solid variant of papillary renal cell carcinoma in adults. In challenging cases, immunohistochemistry and FISH techniques are helpful. With immunohistochemistry, metanephric adenomas usually label for WT1 and CD57 and are characteristically negative for CK7 and AMACR [5]. FISH can be used for analyzing chromosomes 7, 17 and Y. Metanephric adenoma lacks the gains of chromosome 7 and 17 and losses of Y that are typical of papillary renal cell carcinoma [6].

Recently, somatic mutation of the *BRAF* (v-raf murine sarcoma viral oncogene homolog B1) oncogene, located on the long arm of chromosome 7, was identified as a common event in metanephric adenomas [7–12]. BRAF is a serine/threonine kinase that plays a critical role in the mitogen-activated protein kinase (*MAPK)* signaling pathway. The V600E mutation, which accounts for the vast majority of *BRAF* alterations, induces phosphorylation of downstream targets leading to constitutive activation of the cascade. The same mutation has been implicated in the development of many tumors, including melanocytic nevi [13] and melanoma [14], papillary thyroid carcinoma [15], pilocytic astrocytoma [16], colonic adenocarcinoma [17], cholangiocarcinoma [18], borderline ovarian cancer [19], pulmonary adenocarcinoma [20], Langerhans cell histiocytosis [21] and hairy cell leukemia [22]. Of note, immunostaining with the VE1 antibody has been reported as reliable for the detection of *BRAF* V600E mutation in several of the above-mentioned neoplasms [23–29]. Regarding metanephric adenomas, only a few studies [8, 9, 11] containing overall only 20 cases, have investigated the use of immunohistochemistry to detect *BRAF* mutation.

In this study, we correlated *BRAF* mutation, detected by molecular analysis, with *BRAF* V600E immunohistochemical staining in a series of 48 metanephric adenomas and 15 epithelial-predominant nephroblastomas. In addition, we sought to identify clinical and histopathological features of metanephric adenomas harboring *BRAF* mutation.

## RESULTS

Of the 48 patients with metanephric adenoma, 31 were female and 17 were male (F:M ratio, 1.8:1). The median age at diagnosis was 54 years (range: 5 to 84 years) and the median size of the tumor was 4 cm (range from 1.1 to 8 cm) (Table 1). Among the 15 epithelial-predominant nephroblastoma patients, 8 were female and 7 were male (F:M ratio, 1.1:1). The median age was 5 years (range, 8 weeks to 41 years). There were 3 tumors that occurred in adult patients (27, 35, and 41 years, respectively), one female and two male (Table 2).

**Table 1:** Clinical and histopathological features of *BRAF*-mutated and *BRAF*-wild-type metanephric adenomas

Characteristic	BRAF mutated	BRAF wild type	P value
Cases, n (%)	41 (85)	7 (15)	
Gender, n (%)			
Male	11 (27)	6 (86)	**0.0055**
Female	30 (73)	1 (14)	
Age median	57	33	**0.014**
Size median	3.9	3.5	0.71
Architecture			
Pseudocapsule	16	2	0.66
Fibrous septa	12	2	0.99
Histologic pattern (range, %)*			
Acini	36 (5-70)	4 (5-25)	**0.003**
Solid areas	27 (5-100)	6 (5-50)	0.41
Tubules	25 (5-60)	5 (10-50)	0.74
Branching tubules	19 (5-40)	5 (5-20)	0.35
Glomeruloid bodies	11 (5-40)	3 (15-30)	0.32
Papillae	12 (5-80)	2 (20-35)	0.91
Stroma			
Hyalinized	26 (5-40)	6 (5-15)	0.94
Edematous	28 (5-30)	2 (15-20)	0.22
Other features			
Psammoma bodies	22	6	0.11
Foamy Histiocytes	3	0	
Immunohistochemistry			
VE1 positive, n (%)	33 (80)	0	
VE1 negative, n (%)	8 (20)	7 (100)	
p16^INK4^ antibody, n (%)			
5-≤20%	12 (29)	1 (14)	0.65
>20%	29 (71)	6 (86)	

*The numbers indicated cases *BRAF* mutated and *BRAF* wild-type with distinct histopathological characteristics. The range of area occupied by each histological pattern was reported as a percentage in parenthesis.

**Table 2:** Clinical, molecular and immunohistochemical features of epithelial-predominant nephroblastoma

Case	Gender	Age (years)	*BRAF* status	VE1	p1
1	M	3	wild-type	0	30% +
2	F	8 weeks	wild-type	0	0
3	F	27	wild-type	0	<5% +
4	F	3	wild-type	0	0
5	F	6	wild-type	0	30% +
6	F	5	wild-type	0	40% +
7	M	3	wild-type	0	0
8	M	2	wild-type	0	0
9	F	5	wild-type	0	0
10	M	41	wild-type	0	5-10% +
11	M	2	wild-type	0	100% +
12	F	5	wild-type	0	0
13	F	6	wild-type	0	0
14	M	12	wild-type	0	40-50% +
15	M	35	wild-type	0	10% +

M: male, F: female, 0: absence of staining.

**Figure 1:**
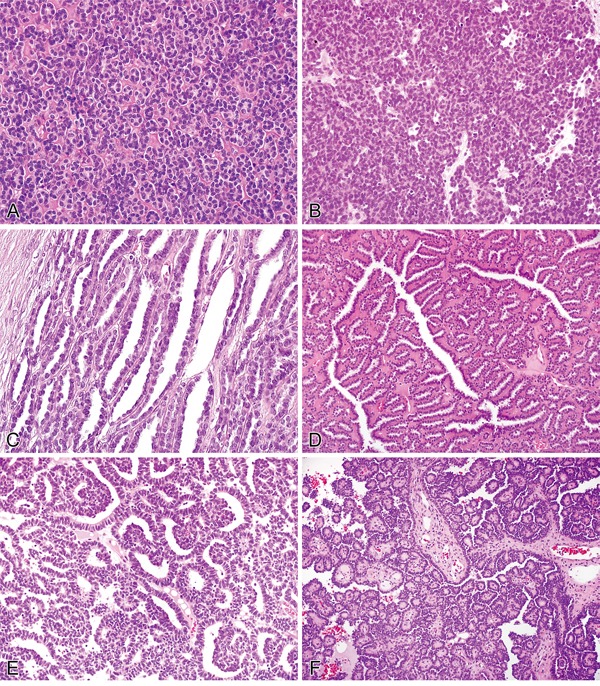
Histopathological features of metanephric adenoma **A**. Acinar pattern. **B**. Solid-like area. **C**. Elongated tubules. **D**. Tubules with branching contours. **E**. Glomeruloid bodies. **F**. Papillary structures.

Microscopic examination of metanephric adenomas showed neoplasms composed of small, uniform and overlapping epithelial cells with scant cytoplasm, inconspicuous nucleoli and essentially no mitotic figures. These cells were arranged in a variety of architectural patterns (Figure 1). Simple tubules, acini and solid patterns were the most common. Papillary structures were present in 14 cases (29%) and were the predominant pattern (>50% of the tumor) in 3. Glomeruloid bodies were present in 14 cases (29%), and branching tubules were present in 24 cases (50%). Eighteen cases (38%) showed a variably thickened and usually discontinuous fibrous pseudocapsule. Fibrous septa that gave a vaguely multinodular appearance were recognized in 14 cases (29%). Twenty-eight tumors (58%) contained psammoma bodies, ranging from isolated and scattered to numerous. A few foamy macrophages were seen in only 3 cases (6%). A stromal component was virtually absent in 4 cases (8%). The remaining cases demonstrated edematous or hyalinized stroma or both (range from 5% to 40% of tumor volume).

*BRAF* V600E mutations were identified in 41 of 48 cases (85%) and none of the other *BRAF* mutation variants was detected. Of these, 30 patients were women and 11 were men (F:M, 2.7:1). The median age was 57 years (range from 5 to 84 years) and the greatest dimension ranged from 1.4 to 8 cm (median = 3.9 cm). Among the 7 *BRAF*-wild-type cases, there was a striking male predominance (F:M 1:6) (p=0.0055), and the patients tended to be younger (median 33, range from 10 to 74) (p=0.014). Tumor size in *BRAF*-wild-type cases (median = 3.5, range from 1.1 to 6.5) was similar to the *BRAF*-mutated cases (p=0.71). Most BRAF-mutated cases exhibited a predominantly acinar architecture (p=0.003). Among 5 cases without this unique histologic feature, 4 were composed mostly of tubules and one mostly of papillae. The other morphological features were not associated with *BRAF* mutational status. None of epithelial-predominant nephroblastomas had *BRAF* mutation.

**Figure 2:**
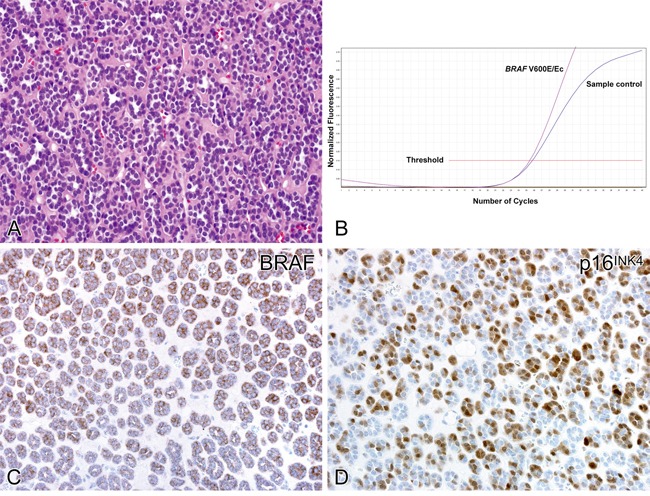
Molecular and immunohistochemical findings in metanephric adenoma Metanephric adenoma predominantly composed of acini harboring *BRAF* mutation **A**. Detection of *BRAF* V600E/Ec mutation **B**. Fluorescence is detected during cycling for both the sample (purple) and sample control (blue). A ≤7.0 difference between the crossing threshold cycles is an acceptable cutoff for a positive V600E/Ec result. The calculated delta CT value of these samples (0.34) demonstrates the detection of V600E/Ec *BRAF* mutation in relation to the sample control. The same case showed a strong cytoplasmic positivity for BRAF VE1 staining **C**. and strong nuclear expression of p16^INK4^ immunolabeling **D**.

There was positive cytoplasmic immunolabeling for VE1 antibody in 33 of 41 (80%) metanephric adenomas with *BRAF* mutation (Figure 2). All VE1 immunostaining positive cases showed *BRAF* V600E mutation, detected by Qiagen BRAF RGQ PCR kit. No nuclear staining was detected in any case. All cases of epithelial-predominant nephroblastoma were completely negative, correlating with their *BRAF* wild-type status. Positive immunolabeling for p16^INK4^ was detected in all metanephric adenomas (range from 5% to 100% of cells), which manifested as nuclear or cytoplasmic staining or both. On the other hand, p16^INK4^ immunostaining was demonstrated in 8 of 15 (53%) epithelial-predominant nephroblastomas.

The histopathological characteristics, molecular and immunohistochemical results are detailed in Table 1 and in Table 2.

## DISCUSSION

*BRAF* is an oncogene that normally functions as a regulator of cell division and differentiation through its role in the MAP kinase pathway. Mutations in this gene, which lead to constitutive activation of downstream signaling within this pathway, were most famously implicated in the development of melanoma [14]. Several specific pro-oncogenic mutations of *BRAF* have since been identified and implicated in the development of a variety of solid and hematopoietic neoplasms [14, 30–32]. The vast majority, however, are characterized by a thymine-to-adenine transversion at exon 15, which results in an amino acid substitution of valine (V) for glutamic acid (E) at codon V600 (V600E) [14]. It is worth noting that clinical trials have recently emerged proposing targeted therapy for nonmelanoma cancers harboring *BRAF* mutations, demonstrating the important role of *BRAF*-testing [33].

Attention to the association of *BRAF* mutation with metanephric adenoma has been drawn by a few case reports and small series [7, 8, 10, 11, 34]. The current study, which tested 48 cases of metanephric adenoma for *BRAF* mutation, is the largest of its kind. Molecular testing demonstrated that 85% of these tumors harbored a *BRAF* mutation; a number in line with the findings of Choueiri et al. [7], who described this mutation in 26 of 29 cases (89%), but somewhat lower than the remaining cases in the literature, which cumulatively yielded a mutated *BRAF* in 22 of 24 cases (92%) (Table 3). All the *BRAF*-mutated cases in this study carried the same V600E mutation, which again is in keeping with the findings of almost all prior studies [7, 9–11, 34]. Of note, Udager et al. [8] identified *BRAF* V600D in 2 of 10 *BRAF*-mutated metanephric adenomas. The current study, which looked for the most common variants, including the V600D, did not find this or any other *BRAF* mutation variant. In this study we also provided clinical and morphologic features characteristic of each metanephric adenoma subset. We found that *BRAF*-mutated cases were associated with older age whereas *BRAF* wild-type metanephric adenomas presented earlier. Consistent with our findings, Choueiri et al. showed increased age (55 vs 33 years) in patients harboring a *BRAF* mutation [7]. A correlation between *BRA*F mutation status and tumor size has been proposed [7], though the current study did not show any size difference, a discrepancy that probably resulted from the limited number of *BRAF*-wild-type cases presented in the prior study. With regards to gender, this is the first study to demonstrate a strong male predominance in metanephric adenomas that are *BRAF* wild-type. Other studies have shown *BRAF* mutation in 3 of 3 and 4 of 5 male subjects respectively [7, 8], however the present study, which included 17 males, is the largest to test for *BRAF* in a male cohort. Also, we included 4 pediatric cases (<12 years old) in our series. As in adults, *BRAF* mutation has been reported in pediatric metanephric adenomas [11]. For the first time, we have outlined distinct morphological features characteristic of *BRAF*-mutated and *BRAF*-wild-type metanephric adenomas. There was a predominance of acinar architecture (p=0.003) associated with *BRAF-*mutated metanephric adenomas. The combination of solid architecture with psammoma bodies and background hyalinized stroma was found to occur with greater frequency in *BRAF*-wild-type cases, but the difference did not reach statistical significance.

**Table 3:** Metanephric neoplasms harboring *BRAF* mutations: literature review

Source, yr	Number of cases	Median age (year, range)	Gender	Tumor size(cm, range)	BRAF mutation	Type of mutation	VE1 positivity	Diagnosis
Choueiri et al., 2012	29	54 (25-78)	26F 3M	2.7 (1.2-7)	26 (89%)	V600E	NA	MA
Dadone et al., 2013	1	61	F	3.5	1 (100%)	V600E	NA	MA
Pinto et al., 2015	6	52	6F	2.6	6 (100%)	V600E	6 (100%)	MA
Udager et al., 2015	11	45 (16-84)	6F 5M	2.7 (1.3-5.1)	10 (91%)	V600E (8) V600D (2)	8 (88%)	MA
Chami et al., 2015	3	9 (4-10)	1F 2M	NA (2-4.5)	2 (67%)	V600E	2 (100%)	MA
	1	4	1M	3.1	1 (100%)	V600E	1 (100%)	MAF
Mangray et al., 2015	1	10	F	1.1	1 (100%)	V600E	1 (100%)	MAF
	3	NA	NA	NA	3 (100%)	V600E	NA	MA
Current study	48	54 (5-84)	31F 17M	4 (1.1-8)	41 (85%)	V600E	33 (80%)	MA

MA metanephric adenoma; MAF metanephric adenofibroma; M male; F female; NA not available.

The presence of a specific and consistent mutation implies a potential role for immunohistochemistry, with the VE1 antibody, as a surrogate for molecular testing, particularly in instances where limited tissue is available or the molecular method for *BRAF* mutation detection is not accessible. Several studies have demonstrated excellent concordance between immunostaining and mutation status in a variety of other neoplasms [25–29]. In the current study, 33 of 41 *BRAF*-mutated cases were positive by immunohistochemistry. Despite it having been speculated that VE1 would be valuable diagnostically [9], this study demonstrated that VE1 antibody is a very specific (100%) but less sensitive (80%) marker for identifying *BRAF*-mutated metanephric adenomas. Moreover, a subset (15%) of metanephric adenomas does not have *BRAF* mutation, prompting again careful use as a diagnostic tool. None of epithelial-predominant nephroblastomas was positive for *BRAF* mutation using either molecular or immunohistochemistry methods in current study. Previous investigations also found that nephroblastomas were negative for *BRAF* mutation by molecular assays [34, 35].

Another aspect of the *BRAF* V600E mutation is its ability to act not only as an oncogene, but paradoxically, to induce cellular senescence. This has been well studied and documented in various *BRAF*-driven neoplasms [36–41]. One of the major markers shown to identify *BRAF*-induced senescence is p16^INK4^ [36]. In light of the indolent clinical course and high frequency of *BRAF* mutation in metanephric adenomas, all cases were stained with the p16^INK4^ antibody. We found that every metanephric adenoma was positive for this marker. The fact that even *BRAF*-wild-type tumors also exhibited positive staining with p16^INK4^ suggests that mechanisms for oncogene-induced senescence independent of *BRAF* mutation exist. In addition, approximately half of the epithelial-predominant nephroblastomas were positive for p16^INK4^, illustrating again that alternative modes of senescence induction may be involved. Interestingly, p16^INK4^ expression has been shown to correlate with good prognosis in nephroblastoma [42]. The mechanism by which some nephroblastomas evade the senescence pathways remains unknown [43].

In summary, we have identified distinct clinicopathologic patterns associated with *BRAF*-mutated metanephric adenoma. These include older age, female predominance, and the presence of a prominent acinar component. A subset of *BRAF*-mutated metanephric adenomas (20%) was not detected by VE1 immunostaining. p16^INK4^ immunostaining was uniformly positive in all metanephric adenomas.

## MATERIALS AND METHODS

### Patients and samples

Forty-eight cases of metanephric adenoma and fifteen cases of epithelial-predominant nephroblastoma were collected from participating institutions. For each case of metanephric adenoma, the following morphologic features were recorded: the presence or absence of a pseudocapsule, foamy histiocytes, and fibrous septa; the relative proportion of stroma and its being either edematous or hyalinized; the presence and quantity of psammoma bodies; and the architectural patterns. With respect to architecture, specific patterns were recognized including tubules with or without complex branching, acini, glomeruloid structures (short, rounded papillae projecting into small cysts), solid-like (tightly packed acini with overlapping nuclei), and papillary. When present, the proportion of the tumor made up of each of these patterns was recorded as a percentage. A pattern that was greater than 50% within the tumor was considered predominant. This research was approved by the Institutional Review Board.

### Immunohistochemistry

Immunohistochemical analysis was performed on all cases utilizing the VE1 antibody, which recognizes the *BRAF* V600E mutant protein (Spring Bioscience, Pleasanton, CA, USA), on whole tissue sections. *BRAF* V600E-mutated melanoma tissue was stained concurrently to serve as a positive control. Cytoplasmic staining was scored as 0 (negative), 1+ (weak), 2+ (moderate) or 3+ (strong) [25]. A positive result required both cytoplasmic staining in >10% of tumor cells and moderate to strong intensity, as previously described [44–46]. In addition, immunohistochemical staining for p16^INK4^ (CINtec^®^, Roche, Germany) was performed on whole sections for each case, and the percentage of positive-staining tumor cells was recorded.

### High resolution melting test for BRAF mutational analysis

The *BRAF* mutations were analyzed using real-time PCR-high resolution melting test. Areas of tumor in each case designated for testing were circled on hematoxylin and eosin-stained slides by a pathologist (LC). The DNA extractions were run using the BRAF RGQ PCR Kit (Qiagen, Valencia, CA), designed to detect somatic mutations of the *BRAF* gene using real-time polymerase chain reaction with the Rotor-Gene Q 5plex HRM instrument. DNA concentrations were analyzed by the NanoDrop ND-1000 Spectrophotometer (NanoDrop Technologies, Wilmington, DE). Using ARMS (Amplification Refractory Mutation System) and Scorpions technologies, the BRAF RGQ PCR Kit detects mutations at codon 600 of the *BRAF* oncogene against a background of wild type genomic DNA. The specific mutations detected by this assay are V600E/V600E complex (V600E/Ec), V600D, V600K, and V600R. All procedures were performed according to the manufacturer's protocol.

### Statistical analysis

Fisher's exact test was used to compare categorical data for clinicopathologic characteristics between *BRAF*-mutated and *BRAF*-wild-type subgroups and Student's t test to compare continuous data. All *P* values were based on a two-sided hypothesis.
